# A sequential Monte Carlo framework for haplotype inference in CNV/SNP genotype data

**DOI:** 10.1186/1687-4153-2014-7

**Published:** 2014-04-24

**Authors:** Alexandros Iliadis, Dimitris Anastassiou, Xiaodong Wang

**Affiliations:** 1 Department of Electrical Engineering, Center for Computational Biology Bioinformatics and Columbia University, New York, NY 10027, USA

## Abstract

Copy number variations (CNVs) are abundant in the human genome. They have been associated with complex traits in genome-wide association studies (GWAS) and expected to continue playing an important role in identifying the etiology of disease phenotypes. As a result of current high throughput whole-genome single-nucleotide polymorphism (SNP) arrays, we currently have datasets that simultaneously have integer copy numbers in CNV regions as well as SNP genotypes. At the same time, haplotypes that have been shown to offer advantages over genotypes in identifying disease traits even though available for SNP genotypes are largely not available for CNV/SNP data due to insufficient computational tools. We introduce a new framework for inferring haplotypes in CNV/SNP data using a sequential Monte Carlo sampling scheme ‘Tree-Based Deterministic Sampling CNV’ (TDSCNV). We compare our method with polyHap(v2.0), the only currently available software able to perform inference in CNV/SNP genotypes, on datasets of varying number of markers. We have found that both algorithms show similar accuracy but TDSCNV is an order of magnitude faster while scaling linearly with the number of markers and number of individuals and thus could be the method of choice for haplotype inference in such datasets. Our method is implemented in the TDSCNV package which is available for download at http://www.ee.columbia.edu/~anastas/tdscnv.

## Introduction

Copy number variations (CNVs) are a form of a structural genomic variation referring to duplications and deletions of DNA segments larger than 1 kilobase in size. CNVs are abundant in the human genome, and it is estimated that they can occupy as much as 4% to 6%.

Recently, large-scale genome-wide studies have shed light in many aspects and characteristics of CNVs providing unique insights into the origins, mechanisms, formation, and population genetics of CNVs [1-3]. At the same time, CNVs have been associated with complex traits unexplained by recent genome wide association studies (GWAS) [2] and are believed to make a substantial contribution in uncovering the mechanisms and etiology of disease phenotypes that result from complex patterns of inheritance [2,4].

A variety of techniques exist for CNV detection. Initially, experimental studies have been performed primarily by array CGH, but lately due to improved resolution and genome coverage of genotyping arrays, a number of methods have been developed relying on whole-genome single-nucleotide polymorphism (SNP) genotyping arrays which offer a more sensitive approach and are more suitable for high-resolution CNV detection. As a result, there is currently simultaneously information on the integer copy number (CN) genotypes along a CNV region and on SNPs outside these regions, in which we will refer in the following as CNV-SNP genotypes.

For diploid organisms, theoretical and empirical arguments have been made for the use of haplotypes as opposed to genotypes. It has been shown that the study of haplotypes can improve the power of detecting associations with diseases, and a variety of methods exist in the literature that use haplotypes to detect causal relationships between a genetic region and a phenotype. Furthermore, haplotypes enable unique insides in the study of populations and are required for many population genetics analyses. Specifically, methods for inferring selection [5] for studying recombination [6,7] as well as historical migration [8,9] build their subsequent analysis on existing haplotype data.

The statistical determination of haplotype phase from genotype data is thus potentially very valuable if the estimation can be done accurately and has received an increasing amount of attention over recent years. A number of well-known algorithms have been developed based on coalescent theory [10], imperfect phylogeny [11], Markov chain Monte Carlo [10,12], Gibbs sampler [13], hidden Markov models [14], expectation minimization algorithm [15], etc. However, only recently, this problem has drawn attention when haplotypes are inferred in a CNV-SNP region.

If we focus within a specific CNV region in a sample of individuals and assume that the ploidy is fixed for each individual along the region, then the problem of inferring the haplotypes is identical to the problem of inferring the haplotypes in polyploid organisms or estimating haplotypes from pooling data. A number of algorithms have been proposed for frequency estimation and inference on these settings, and not surprisingly, many have been applied to the associated CNV haplotype inference problem described above.

Apart from the previous scenarios, a number of methodologies have been specifically developed and tailored for CNV data. Kato et al. [16] have developed a methodology MOCSphaser based on the EM algorithm to assign copy numbers in their respective chromosomes in regions that include CN and SNPs. A core limitation of MOCSphaser as described above is that it takes into consideration only the total CN and not the alleles themselves, assigning on each chromosome a raw CN. As a consequence, even though it provides information about the total copies on a chromosome that could be potentially useful, it does not provide information on the diplotypes themselves.

Another algorithm recently proposed by Kato et al. [17], CNVphaser uses an EM approach to perform inference. The core limitation of that method is that the inference is performed within a CNV region and that the ploidy is considered fixed for an individual within the region. To address these problems and thus enabling the phasing of regions where the ploidy of an individual varies along the region and each individual can have different breakpoints, Su et al. [18] suggested polyHap(v2.0) in which they extended the functionality of their original methodology for pooling data [19]. In their study, they discern the phasing within a CNV into non-internal phasing in which the CNV in a chromosome is inferred as a diplotype and internal phasing in which the specific haplotypes comprising the CNV in a chromosome are further identified. We will use these definitions in our current work.

In their algorithm, Su et al. use an HMM methodology that has separate emission states for the internal and non-internal phasing. They treat the transition between states conceptually in a hierarchical two-level model where the first level is for the transition among CN states and the second for the transition among the haplotype states given the CN states. polyHap(v2.0) is the only currently available method that can phase complex CNV regions by allowing arbitrary changes of CN within individuals and along the genomic sequence.

In this paper, we propose a related new sequential Monte Carlo algorithm for haplotype phasing of CNV-SNP data. In our method, samples are processed sequentially and our method scales linearly with the number of samples as well as the number of individuals. We demonstrate that using our methodology, we can achieve state-of-the-art performance while our method is an order of magnitude faster than polyHap (v2.0).

## Methods

The structure of this section is as follows. In the beginning of the section, we introduce some notation that we will use throughout the remaining manuscript. In the subsections that follow, we present the modified version of our TDS methodology for the case of CNV-SNP data. For completeness, we develop again our framework in detail as presented in [20,21]. We first present some modeling results for the prior and posterior distributions for the population haplotype frequencies given the observed data. We then present the TDS methodology for the cases of known population frequencies and subsequently extend it to the case of unknown frequencies. In the derivation of the later, we use the previously derived results for the prior and posterior distributions for the haplotype frequencies. We end the exposition of our method by deriving the state update equations for the ‘Tree-Based Deterministic Sampling CNV’ (TDSCNV) estimator and presenting the modified partition-ligation procedure adjusted for the CNV-SNP dataset scenario. In the end of the section, we describe the procedure for creating the datasets which we have used in the ‘Results’ section to evaluate our methodology.

### Definitions and notation

Suppose we are given a set of CNV-SNP genotypes on *L* diallelic loci. We denote the two alleles at each locus by 0 and 1. In the following, we will use the counts of allele 1 as the provided measurement for each allele on each sample. In our method, we allow in a specific position a single amplification or deletion. Therefore, if we are within a CNV region in a chromosome, the allele counts could range from 0 to 2 but could range from 0 to 1 outside these regions.

Suppose that we have *T* individuals and we denote $c_{t}=\left\{c_{t}^{1},\ldots ,c_{t}^{L}\right\}$ to be the observed genotype of the *t*-th sample where $c_{t}^{i}\in \left\{0,1,2,3,4\right\}$ are the observed counts on the *i*th position. Suppose also that *C*_*t*_ = {*c*_1_, …, *c*_*t*_} is a set of individuals up to and including individual *t* and let *C* denote the full set of individuals.

In terms of haplotypes, we make an initial distinction in the values that alleles take in internal and non-internal phasing. The framework that follows however will be described generically and will be the same in both cases.

For non-internal phasing, our purpose is to infer haplotypic phase on diploid chromosomes as we are interested in the total copies of an allele at a specific position on a chromosome. Therefore, the possible values for an allele at each position are {−,0,1,01,00,11}. On the contrary for internal phasing, we infer haplotypic phase on polyploid chromosomes and the possible alleles at each position are {−,0,1}.

For individual *t*, we denote the haplotypes occurring in that individual as *h*_*t*_. In the case of non-internal phasing, *h*_*t*_ = {*h*_*t*,1_, *h*_*t*,2_}. For internal phasing, *h*_*t*_ = {*h*_*t*,1_, …, *h*_*t*,*p*_}, where *p* is the ploidy of the organism, and *p* ∈ {1, 2, 3, 4} as in our methodology, we only consider a single deletion or a single amplification. Therefore, for the case of non-internal phasing *h*_*t*,1_, *h*_*t*,2_ are strings of length *L* in which *h*_*t*,*i*,*j*_ ∈ {−, 0, 1, 01, 00, 11} and for internal phasing, *h*_*t*,*i*_ are strings of length *L* in which *h*_*t*,*i*,*j*_ ∈ {−, 0, 1}.

We further denote *H*_*t*_ = {*h*_1_, …, *h*_*t*_}, similarly to *C*_*t*_ as the set of haplotypes for each individual up to and including individual *t*.

Let us also define *z* = {*z*_1_, …, *z*_*M*_} as the set containing all haplotype vectors of length *L* that are consistent with any genotype in the set C. To obtain Z from the given dataset C, we first enumerate for each *c*_*i*_ the subset $\psi_{i}=\left\{h_{i}^{1},\ldots ,h_{i}^{Y}\right\}$*i* = *1*,…,*T* that contains all possible haplotype assignments which are consistent with *c*_*i*_. The set Z is then given simply as $Z=U_{i=1}^{T}\psi_{i}$. A set of population haplotype frequencies *θ* = {*θ*_1_, …, *θ*_*M*_} is also associated with the set Z of all possible haplotype vectors, where *θ*_*m*_ is the probability with which the haplotype *z*_*m*_ occurs in the total population. We note here once again that we have given the definitions of Z and *θ* generically for both internal and not internal phasing, respectively.

### Prior and posterior distribution for *θ*

Assuming random mating in the population, it is clear that the number of each unique haplotype in *H* is drawn from a multinomial distribution based on the haplotype frequency *θ*[22]. This leads us to the use of the Dirichlet distribution as the prior distribution for *θ* so that *θ* ~ *D*(*ρ*_1_, …, *ρ*_*M*_). It is well known in Bayesian statistics that the Dirichlet distribution is the conjugate prior of the multinomial distribution. This implies in our case that if we assume that the prior distribution for *θ* is Dirichlet and we draw haplotypes based on their frequencies (multinomial distribution), then the posterior distribution for *θ* is again a Dirichlet distribution. We prove this fact below.

(1) $$\begin{array}{l} p\left(\theta |C_{t},H_{t},Z\right)\propto p(c_{t}|h_{t}=\left(h_{t,1},\ldots ,h_{t,p}\right),\theta ,C_{t-1},H_{t-1})p\\ \;\times (h_{t}=(h_{t,1},\ldots ,h_{t,p})|\theta ,C_{t-1},H_{t-1},Z)p(\theta |C_{t-1},H_{t-1})\\ \;\propto p(h_{t}=\left(h_{t,1},\ldots ,h_{t,p}\right)|\theta ,Z)p(\theta |C_{t-1},H_{t-1},Z)\\ \;\propto \prod_{i=1}^{p}\theta_{h_{t,i}}\prod_{m=1}^{M}\theta_{m}^{\rho_{m}\left(t-1\right)-1}\propto \prod_{m=1}^{M}\theta_{m}^{\rho_{m}\left(t-1\right)-1+\sum_{i=1}^{p}I\left(z_{m}-h_{t,i}\right)}\\ \;\propto D(\rho_{1}\left(t-1\right)+\sum_{i=1}^{p}I\left(z_{1}-h_{t,i}\right),\ldots ,\rho_{M}(t-1)+\sum_{i=1}^{p}I\left(z_{M}-h_{t,i}\right)) \end{array}$$

where we denote *ρ*_*m*_(*t*) *m* = *1*,…,*M* as the parameters of the distribution of *θ* after the *t*-th pool and *Ι*(*z*_*m*_ − *h*_*t*,*i*_) is the indicator function which equals 1 when *z*_*m*_ − *h*_*t*,*i*_ is a vector of zeros, and 0 otherwise. We note here once again that the number of haplotypes (i.e., the index *p* in the assignment) depends on the phasing and is 2 for non-internal phasing while it ranges for internal phasing. Furthermore, in the previous calculations for *θ*, for each genotype vector, we only consider haplotype configurations that are consistent with that genotype.

We have shown that the posterior distribution for *θ* is also Dirichlet with parameters as given in (1) and depends only on the sufficient statistics, *T*_*t*_ = {*ρ*_*m*_(*t*), 1 ≤ *m* ≤ *M*} which can be easily updated based on *T*_*t* − 1_, *h*_*t*_, *c*_*t*_ as given by (1) i.e., *T*_*t*_ = *T*_*t*_(*T*_*t* − 1_, *h*_*t*_, *c*_*t*_).

### TDS estimator with known system parameters *θ*

Similar to traditional sequential Monte Carlo (SMC) methods, we assume that by the time we have processed genotype *c*_*t*-1_, we have a set of K potential solution streams (commonly termed as ‘particles’) $H_{t-1}^{\left(k\right)}$ (*k* = 1, …., *K*) each associated with its corresponding weight $w_{t-1}^{\left(k\right)}$, as $\left\{\left(H_{t-1}^{\left(k\right)}|w_{t-1}^{\left(k\right)}\right),k=1,\ldots ,K\right\}.$

At point *t-*1, we approximate the real continuous distribution *p*(*H*_*t* − 1_|*C*_*t* − 1_) as a discrete distribution as follows:

(2) $$\hat{p}\left(H_{t-1}|C_{t-1}\right)=\frac{1}{W_{t-1}}\sum_{k=1}^{K}w_{t-1}^{\left(k\right)}I\left(H_{t-1}-H_{t-1}^{\left(k\right)}\right)$$

where

$$W_{t-1}=\sum_{k=1}^{K}w_{t-1}^{\left(k\right)},$$

and *I* (●) is the indicator function such that I (*x* − y) = 1 for *x* = *y* and I (*x* − y) = 0 otherwise.

Processing the next individual *t*, we would like to make an online inference of the haplotypes *H*_*t*_ based on the genotypes *C*_*t*_. From Bayes' theorem, we have $p_{\theta }\left(H_{t}|C_{t}\right)\propto p_{\theta }(c_{t}|H_{t},C_{t-1})p_{\theta }(H_{t}|C_{t-1})\propto p_{\theta }(c_{t}|H_{t},C_{t-1})p_{\theta }(h_{t}|H_{t-1},C_{t-1})p_{\theta }(H_{t-1}|C_{t-1})\propto p_{\theta }\left(h_{t}|H_{t-1},C_{t-1}\right)p_{\theta }(H_{t-1}|C_{t-1})$ where for our purposes, we only consider haplotype assignments for individual *t* that are compatible to its observed genotype.

Assume further that there are *K*^ext^ such assignments. From previous relationships, if we knew the system parameters *θ*, we would be able to approximate the distribution of *p*_*θ*_(*H*_*t*_|*C*_*t*_) as follows:

(3) $${\hat{p}}_{\theta }\left(H_{t}|C_{t}\right)=\frac{1}{W_{t}^{\mathrm{ext}}}\sum_{k=1}^{K}\sum_{i=1}^{\mathrm{Kext}}w_{t}^{\left(k,i\right)}I\left(H_{t}-\left[H_{t-1}^{\left(k\right)},h_{i}^{\left(i\right)}\right]\right)$$

where $\left[H_{t-1}^{\left(k\right)},h_{t}^{\left(i\right)}\right]$ represents the vector obtained by appending the element $h_{t}^{\left(i\right)}$ to the vector $H_{t-1}^{\left(k\right)}$ and $W_{t}^{\mathrm{ext}}=\sum_{i,k}w_{t}^{\left(k,i\right)}$ with

$$w_{t}^{\left(k,i\right)}\propto w_{t-1}^{\left(k\right)}p_{\theta }\left(c_{t}|h_{t}=i\right)p_{\theta }\left(h_{t}=i|H_{t-1}^{\left(k\right)}\right).$$

### TDS estimator with unknown system parameters *θ*

However, the system parameters are not known. In our model, we use a Dirichlet distribution, as the prior for *θ* and as shown, we obtain a posterior distribution for *θ* (given *H*_*t*_ and *C*_*t*_) that is Dirichlet and only depends on a set of sufficient statistics.

Using Bayes' theorem and similarly to the previous subsection, we have:

(4) $$\begin{array}{l} p_{\theta }\left(H_{t}|C_{t},Z\right)\propto p_{\theta }(c_{t}|H_{t},C_{t-1})p_{\theta }\\ \;\times (h_{t}|H_{t-1},C_{t-1})p_{\theta }(H_{t-1}|C_{t-1},Z)\propto p_{\theta }\left(H_{t-1}|C_{t-1},Z\right)p_{\theta }\\ \;\times (c_{t}|H_{t},C_{t-1})\int p\left(h_{t}|H_{t-1},\theta ,Z\right)p(\theta |T_{t-1},Z)\mathrm{d\theta}\propto p_{\theta }\\ \;\times \left(H_{t-1}|C_{t-1},Z\right)\int p\left(h_{t}|H_{t-1},\theta ,Z\right)p(\theta |T_{t-1},Z)\mathrm{d\theta} \end{array}$$

where again we only consider haplotype assignments that are compatible with the observed genotype.

Taking into consideration as argued before that if we know the system parameters *θ*, then the *p*(*h*_*t*_|*H*_*t* − 1_, *θ*, *Z*) term represents sampling from a multinomial distribution and that the mean of the Dirichlet distribution with respect to an element *θ*_*k*_ of the vector *θ* is as follows:

$$E\left\{\theta_{k}\right\}=\frac{\rho_{k}}{\sum_{j=1}^{M}\rho_{j}}$$

we have from (4) that:

(5) $$\begin{array}{l} p_{\theta }\left(H_{t}|C_{t},Z\right)\propto p_{\theta }(H_{t-1}|C_{t-1},Z)\int p\left(h_{t}|H_{t-1},\theta ,Z\right)p\\ \;\times (\theta |T_{t-1},Z)\mathrm{d\theta}\propto p\left(H_{t-1}|C_{t-1},Z\right)\int \left(\prod_{i=1}^{{}_{M}}\theta_{k}^{\sum_{i=1}^{p}I\left(z_{k}-h_{t,i}\right)})p\right)\\ \;\times (\theta |T_{t-1},Z)\mathrm{d\theta}\propto p\left(H_{t-1}|C_{t-1},Z\right)\int (\prod_{i=1}^{{}_{M}}\theta_{k}^{r_{k}})\frac{1}{B\left(\rho \left(t-1\right)\right)}\prod_{i=1}^{M}\theta_{i}^{\rho_{i}\left(t-1\right)-1}\mathrm{d\theta}\propto p\\ \;\times \left(H_{t-1}|C_{t-1},Z\right)\frac{B\left(\rho \left(t-1\right)+r\right)}{B\left(\rho \left(t-1\right)\right)}\int \frac{1}{B\left(\rho \left(t-1\right)+r\right)}\prod_{i=1}^{M}\theta_{i}^{\rho_{i}\left(t-1\right)+r_{i}-1}\mathrm{d\theta}\propto p\\ \;\times \left(H_{t-1}|C_{t-1},Z\right)\frac{B\left(\rho \left(t-1\right)+r\right)}{B\left(\rho \left(t-1\right)\right)} \end{array}$$

where $r=\left[\sum_{i=1}^{p}I\left(z_{1}-h_{t,i}\right),\ldots ,\sum_{i=1}^{p}I\left(z_{M}-h_{t,i}\right)\right]\;$ and $B\left(\rho \left(t-1\right)\right)=\frac{\prod_{i=1}^{M}\Gamma \left(\rho_{i}\left(t-1\right)\right)}{\Gamma \left(\sum_{i=1}^{M}\rho_{i}\left(t-1\right)\right)}.$

Assuming that we have approximated *p*(*H*_*t* − 1_|*C*_*t* − 1_) as in (2), we can approximate *p*(*H*_*t*_|*C*_*t*_) using (5) as follows:

$${\hat{p}}^{\mathrm{ext}}\left(H_{t}|C_{t}\right)=\frac{1}{W_{t}^{\mathrm{ext}}}\sum_{k=1}^{K}\sum_{i=1}^{\mathrm{Kext}}w_{i}^{\left(k,i\right)}I\left(H_{t}-\left[H_{t-1}^{\left(k\right)},\left(h_{t,1}^{i},\ldots ,h_{t,p}^{i}\right)\right]\right)$$

where the weight update formula is given by:

(6) $$w_{t}^{\left(k,i\right)}\propto w_{t-1}^{\left(k\right)}\frac{B\left(\rho^{\left(k\right)}\left(t‒1\right)+r\right)}{B\left(\rho^{\left(k\right)}\left(t‒1\right)\right)}$$

where again $r=\left[\sum_{i=1}^{p}I\left(z_{1}-h_{t,i}^{j}\right),\ldots ,\sum_{i=1}^{p}I\left(z_{M}-h_{t,i}^{j}\right)\right]$ and *ρ*^(*k*)^(*t* − 1) is the parameter vector of the assumed Dirichlet prior which represents how many times we have encountered each haplotype in stream *k* in the solutions up to individual *t* − 1.

### Partition-ligation

In the partition phase, the dataset is divided into small segments of consecutive loci and each of the individual blocks is phased separately. To ligate the individual blocks, we have adjusted the original partition-ligation (PL) method for the case of CNV-SNP data.

In our current implementation, to be able to derive all possible solution combinations for each pool genotype efficiently, we have decided to keep the maximum block length to 5 SNPs. Clearly, the more SNPs are included in a block, the more information about the LD patterns we can capture but at the same time, the number of possible combinations increases and becomes prohibitive for more than 5 SNPs. For our experiments in a dataset with *L* loci, we have considered *L/*5 blocks of 5 consecutive loci and the remaining SNPs were treated as a separate block.

The result of phasing for each block is a set of haplotype solutions for each genotype. Two neighboring blocks are ligated by creating merged solutions for each genotype from combinations of the block solutions, each associated with the product of the individual solution weights called the *ligation weight*.

Depending on which haplotypes one from each block are going to be assigned on the same chromosome for each individual, a different number of changes in the ploidy of that individual will occur. In our method, we consider only the assignments that will produce the minimum number of such changes. Therefore, if both haplotypes in any block have the same CN, we examine both alternative assignments but we otherwise ligate solutions that have the same CN. The TDS algorithm is then repeated in the same manner as it was for the individual blocks with the weights of the solutions scaled by the associated ligation weight for that solution.

### Summary of the proposed algorithm

### Dataset creation

Our datasets consisted of SNPs from chromosomes 1 and 2 from HapMap CEU population (HapMap3 release 2 - phasing data). For our purposes, we have considered only the parents in each trio which are the unrelated individuals in our dataset thus resulting in a total of 88 individuals. We have initially filtered out SNPs with minor allele frequencies less than 5%, and we have then considered non-overlapping datasets with a fixed number of SNPs. To create artificial CNV regions within each dataset, we have used the following procedure.

First, in each dataset, we have found all the different haplotypes appearing in the dataset. In order to retain as much of the LD structure and also the property that most of the CNVs could be flagged by neighboring SNPs [2], we have randomly replaced specific areas of randomly chosen haplotypes with a CNV haplotype. To perform that procedure, we randomly selected haplotypes based on their frequency in the population and modified them inserting CNV regions sequentially as follows. Each position was considered as the beginning of a CNV region with a probability of 0.1. For each position flagging the beginning of a CNV, we assigned the length of the CNV region uniformly between three to eight SNPs. We then progressed along the haplotype from the end of the CNV region in a similar fashion until we reached the end of a given haplotype.

## Results

### Measurement of phasing accuracy

We have used a number of different measures to evaluate the performance of our methodology. First, the switch error rate [23,24] is defined as the percentage of switches among all possible switches in haplotype orientation used to recover the correct phase in an individual.

In the case of a small number of loci where haplotype vectors can be expected to be reconstructed exactly, we have used two figures of merit namely the × ^2^ and *l*_1_ distance to evaluate the accuracy of frequency estimation. Suppose that *f* are the predicted haplotype frequencies from an algorithm and *g* are the gold standard population level haplotype frequencies. The × ^2^ distance between the two distributions is simply the result of the × ^2^ statistic, i.e., $\chi^{2}\left(f,g\right)=\sum_{i=1}^{d}{\left(f_{i}-g_{i}\right)}^{2}/g_{i}$ where *d* is the number of gold standard haplotypes whereas the *l*_1_ distance between the two distributions is defined as $l_{1}\left(f,g\right)=\sum_{i=1}^{d}|f_{i}-g_{i}|$[25].

### Switch error rate

We have compared the performance of our method with polyHap(v2.0) for haplotypic phase inference using the switch error rate. In this section, the evaluation was done on non-internal haplotypes. In the evaluation of the switch error rate, we consider only CN and SNP positions that are ambiguous. For a marker genotype to have ambiguous phasing, there should be at least two alternative orientation assignments. As an example, all 3CN genotypes are ambiguous positions. This is easy to see, as the choice alone of the chromosome that would have the duplication creates two distinct possible assignments.

The performance of our method when considering the full set of individuals in each dataset is shown in Table 1. We have considered three marker sizes namely 30, 50, and 100 markers. For each marker size, we have simulated 100 datasets and the result presented is the average error rate on these 100 datasets. We can see that for 30 and 50 markers, our method was marginally better than polyHap(v2.0), whereas for the 100 marker datasets, it was marginally worse.

**Table 1:** **Switch error rate Switch error rates for non**-**internal phasing**

	**Number of markers**		
	**30**	**50**	**100**
TDSCNV	0.115	0.127	0.14
polyHap(v2.0)	0.128	0.135	0.138

The switch error rate presented for each number of markers is the average on 100 datasets.

**Figure 1:**
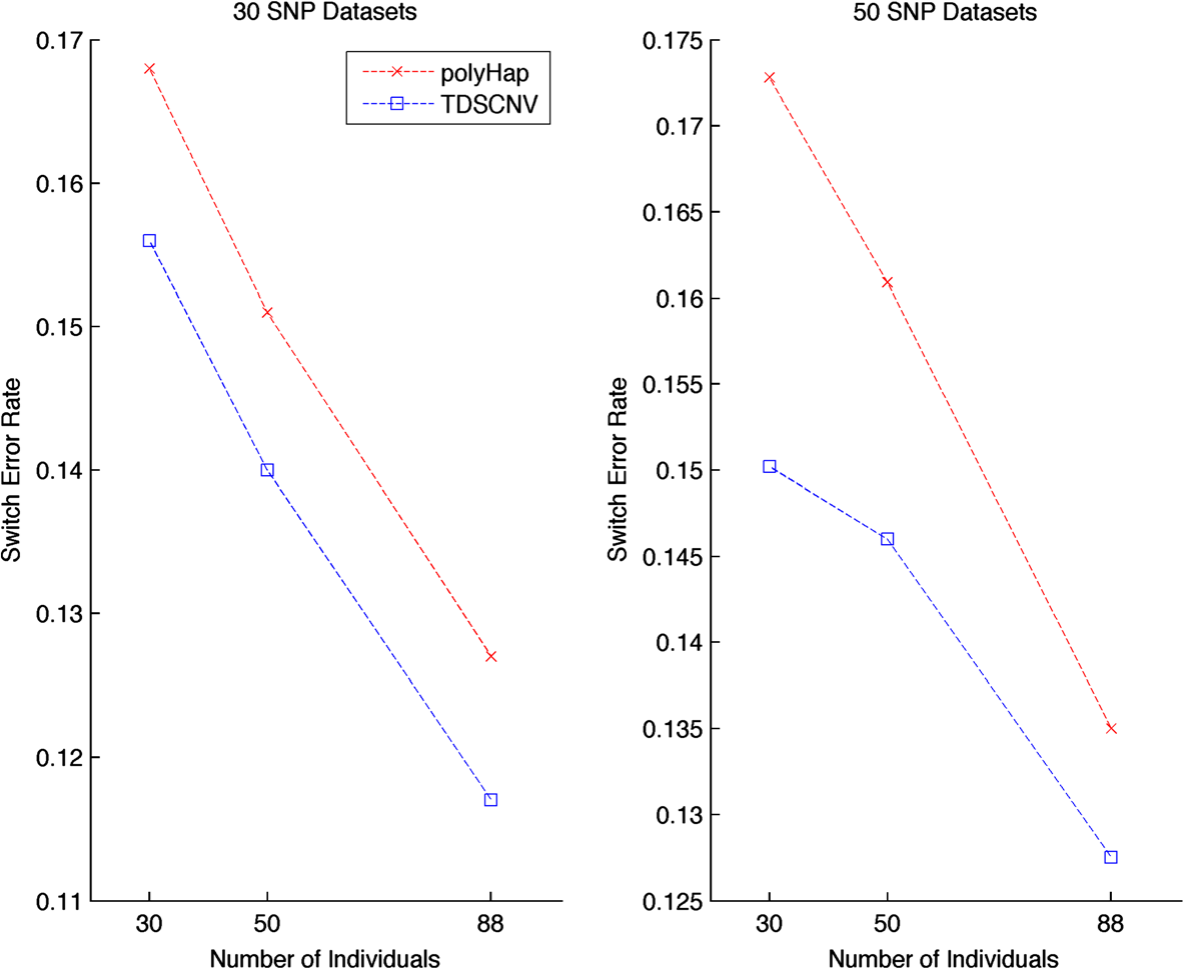
**Switch error rate.** Estimating the switch error rate for non-internal phasing on datasets having a varying number of individuals with polyHap(v2.0) and TDSCNV.

We further demonstrate the accuracy of our approach when ranging the number of individuals in each dataset. The results for a fixed number of 30 and 50 markers are shown in Figure 1. As expected, the performance for both methods improves with increasing number of individuals per dataset.

Finally, we have broken down and calculated the switch error rates based on the CN of the ‘from’ and ‘to’ sites as shown in Table 2. Similarly, to Su et al., we observe the highest switch error rates appearing when the transitions happen between different CNs.

**Table 2:** **Switch error rate for non**-**internal phasing according to the CN of the respective consecutive ambiguous markers**

	**CN on second site**	
**CN on first site**	**1**	**2**
1	0.117	0.227
2	0.229	0.012

### Haplotype frequency estimation

**Figure 2:**
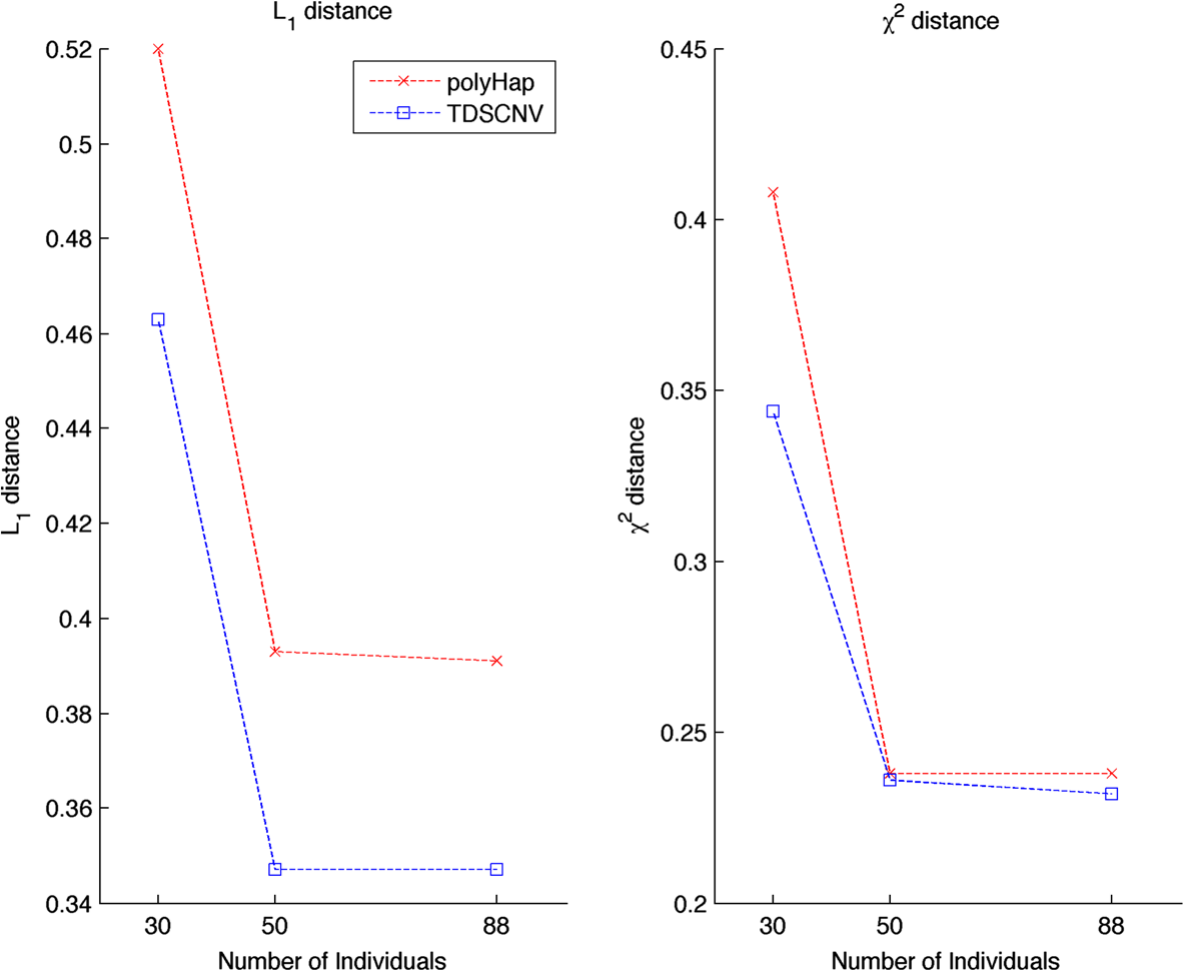
**Frequency estimation.** Estimating the × ^2^ and *l*_1_ distance on datasets having a varying number of individuals with polyHap (v2.0) and TDSCNV.

We have examined the accuracy of our method and compared it against polyHap(v2.0) on datasets of 8 and 10 markers in which individuals had a fixed ploidy. We have evaluated two appropriate figures of merit as described above, the × ^2^ and *l*_1_ distance. We should note here that in order to determine how good frequency estimations with a given method are, a small number of markers should be used. The reason is that for a large number of markers, it would be unlikely that the exact same haplotypes would appear or reconstructed with appreciable frequency. The results for both figures of merit on an increasing number of individuals are shown in Figure 2. Our method demonstrates superior performance for both figures of merit, and again as expected, both methods produced superior performance with an increasing number of individuals.

### Internal phasing

We have further evaluated the performance of our method using the switch error rate inside duplicated regions. In this subsection, the evaluation was done on internal phasing and particularly in duplicated segments of a chromosome as the scope was to detect how good the specific haplotypes comprising the duplicated chromosomal region could be recovered. The switch error rate evaluation within such duplicated regions is exactly the same as the evaluation on a genotype with only SNPs.

We have used the same 100 datasets for each of the three dataset sizes, namely 30, 50, and 100 markers, as in the evaluation of the switch error rate for non-internal phasing described in a previous subsection. We found, as expected, that the results were similar irrespectively of the dataset size, and the average across all datasets was 0.183.

### Timing results

The computational times for the 30, 50, and 100 marker datasets used for the calculation of the switch error rate are displayed in Table 3. We can see that TDSCNV is an order of magnitude faster than polyHap(v2.0) for all marker sizes examined.

**Table 3:** Timing results

	**Number of markers**		
	**30**	**50**	**100**
TDSCNV	2.1	3.7	5.7
polyHap (v2.0)	262.3	431.5	892.1

For each method and each marker size, the computational time is the average time on the 100 datasets used in the switch error rate calculation. Time is given in seconds.

## Discussion

We present an algorithm for haplotypic inference in regions of CNV-SNP genotypes. We compare our method with polyHap(v2.0) on a variety of marker sizes and evaluate the accuracy and computational time of each method. Our method has similar accuracy to polyHap(v2.0) but is an order of magnitude faster in all datasets examined.

In all instances of haplotype inference problems, it becomes increasingly significant that methods are able to incorporate prior knowledge in the form of haplotypes or genotypes from the same population as that from which the target samples were drawn. HapMap is a striking example of such database knowledge that could be used for haplotype inference. Furthermore, it is also important for researchers that samples that are phased at some point in time could be used efficiently for the phasing of samples presented at some later point. Our methodology offers a unique framework that can easily incorporate such prior knowledge. Haplotypes can be introduced in the form of a prior for the counts in the TDSCNV algorithm. From our experience with our framework and as expected, the presence of the extra information will improve the phasing accuracy of the target samples.

## Conclusions

In this paper, we propose a new sequential Monte Carlo algorithm for haplotype phasing of CNV-SNP data. In our method, samples are processed sequentially and our method scales linearly with the number of samples as well as the number of individuals.

To demonstrate the performance of our method, we have compared it against polyHap(v2.0), the only currently available software able to perform inference in CNV/SNP genotypes, on datasets of varying number of markers. We have initially compared the accuracy of both methods for haplotypic phase inference on non-internal haplotypes, on datasets of 30, 50, and 100 markers. We have then examined the accuracy of frequency estimation with both methods on datasets with a small number of markers (8 and 10 markers). Finally, we have evaluated the performance of our methodology inside duplicated regions for internal phasing.

We have found that our method demonstrates comparable or better accuracy than polyHap(v2.0) and at the same time is an order of magnitude faster in all datasets and marker sizes examined while scaling linearly with the number of markers and number of individuals. We therefore believe that our method could be the method of choice for haplotype inference in such datasets.

## Competing interests

All authors declare they have no competing interests.
